# The nitazene epidemic in Estonia: a first report

**DOI:** 10.1093/eurpub/ckaf160

**Published:** 2025-09-23

**Authors:** Katri Abel-Ollo, Mailis Tõnisson, Peep Rausberg, Aime Riikoja, Tarmo Barndõk, Mikk Oja, Gleb Denissov, Don Des Jarlais, Anneli Uusküla

**Affiliations:** Drugs and Addictions Department, National Institute for Health Development, Tallinn, Estonia; Southern Estonian Forensic Medical Department, Estonian Forensic Science Institute, Tartu, Estonia; Institute of Clinical Medicine, University of Tartu, Tartu, Estonia; Department of Chemistry, Estonian Forensic Science Institute, Tallinn, Estonia; Department of Toxicology, Estonian Forensic Science Institute, Tallinn, Estonia; Department of Toxicology, Estonian Forensic Science Institute, Tallinn, Estonia; Drugs and Addictions Department, National Institute for Health Development, Tallinn, Estonia; Department of Registries, National Institute for Health Development, Tallinn, Estonia; School of Global Public Health, New York University, New York, NY, United States; Department of Family Medicine and Public Health, University of Tartu, Tartu, Estonia

## Abstract

Since 2022, Estonia, a north-east European nation of 1.3 million people, has faced challenges with nitazenes, a class of novel synthetic opioids, which present a new threat to public health. The purpose of this article is to provide the timeline of the nitazene epidemic in Estonia, examining the prevalence and health consequences of nitazene use in the country. This case study uses a multifaceted approach. Data sources include administrative statistics, surveillance and research data, national service provision information, and government documentation from 2015 to 2024, with a focus on health consequences from 2019 to 2024. Quantitative data is complemented by qualitative interviews with nitazene users. The number of drug-related deaths in Estonia has more than doubled since 2022 (39 vs. 80 cases), exceeding over 100 cases in 2023. The increasing prevalence of nitazenes from 2022 is confirmed by syringe residue studies and seizure data. Nitazenes are often sold on the drug market, usually with no or limited information to the user about the substance being sold. Users frequently describe the effect of nitazenes as stronger, faster, sharper and more short-lived compared to fentanyl. Harm reduction services have seen increased utilization since 2022, with first responders facing growing challenges linked to the emergence of the nitazene phenomenon. This study provides the first comprehensive description of the nitazene epidemic. Results indicate a need for more evidence-based information on the use of nitazenes and their consequences to effectively address emerging challenges.

## Introduction

The global landscape of illicit drug use has been in a state of constant flux, characterized by an alarming and continuous growth in the number of individuals engaging in such use, coupled with the emergence of new and potent synthetic substances [1]. New substances on the illicit opioid market, characterized by the availability of highly potent and often adulterated substances, present unique challenges in terms of prevention, treatment, and harm reduction. Learning from, and understanding the dynamics of, this evolving crisis is crucial for developing effective strategies to mitigate the negative consequences associated with illicit drug use.

Opioids originate from various sources, ranging from natural poppy plants to potent synthetic derivatives such as fentanyl. Since 2019 in Europe and North America, increasingly potent synthetic opioids, including nitazenes, which are more potent than fentanyl, are being detected [2–5]. Nitazenes (benzylbenzimidazoles), a group of synthetic opioids derived from 2-benzylbenzimidazole, were developed 60 years ago as potential painkillers but were never approved due to their high potency and overdose risk [6]. In 2024, nitazenes have been available on recreational drug markets in the form of powders, counterfeit tablets, or liquids, and have been detected in illicit heroin, fentanyl, and adulterated benzodiazepines and pain medicines, exacerbating the opioid crisis [3]. The use of nitazenes poses a significant threat to public health due to their unknown properties and lack of established treatment protocols [7–9].

The United States of America (USA) has struggled with a synthetic opioid crisis related to fentanyl for the last 10 years, resulting in an alarming increase in drug-related cases of overdose deaths [10]. Since 2022, nitazenes have also been present in some states of the USA but have currently lagged far behind fentanyl [3]. Synthetic opioids play a relatively small role overall in Europe’s drug market but feature prominently in the illicit drug market in the Baltic countries of Estonia and Latvia [11]. For the past 2 decades, Estonia has experienced an unparalleled crisis with synthetic opioids in comparison to the rest of Europe, resulting in the highest overdose death mortality rate [12].

The fentanyl epidemic of 2002–17 in Estonia, including its evolution and harm reduction responses, is comprehensively documented [12, 13]. At the end of 2017, the fentanyl era ended because of disruption of the main criminal networks involved with fentanyl trafficking, resulting in considerably low numbers of drug-related overdose deaths for some years [14]. Since 2019, nitazenes have been present on the Estonian drug market. In 2022, most synthetic opioid seizures in Europe occurred in Estonia and Latvia (primarily nitazenes) and Lithuania (carfentanil) [15]. This article aims to delineate the temporal evolution of the nitazene epidemic in Estonia, examining the increasing presence and health consequences of its use in the country.

## Methods

### Study design and implementation

We conducted a country case study [16, 17] of Estonia, examining the period from 2015 to 2024. Data were drawn from various primary and secondary sources, including administrative statistics, surveillance, and research data, national information on service provision, and government documentation. To understand experiences with nitazenes, we gathered information from two main groups: people who use drugs (semi-structured interviews to gather their first-hand accounts and perspectives), and first responders and treatment providers (the first author collected structured information from these key informants through personal communication via email and phone).

We conducted the case study as a form of knowledge synthesis to address the questions: (i) how has the problem of nitazene use evolved in terms of prevalence and usage patterns in Estonia from 2015 to 2024; and, (ii) what have been the health consequences of nitazene use in Estonia during the period when nitazenes have been present?

### Quantitative data sources

We systematically searched existing data in the field and included diverse sources to examine the nitazenes epidemic in Estonia. We reviewed all available surveillance and research data on illicit drugs, searching for indications of new synthetic opioids or nitazenes. The extracts from administrative databases were requested for the period 2015–24, including the Causes of Death Registry, Estonian Forensic Science Institute seizure data, and Estonian Health Board records (Supplementary Table S1). This multisource approach allowed us to corroborate findings from different angles: supply (seizures), use (syringe residues), and impact (deaths). The consistency of these trends across independent quantitative databases provided a robust and triangulated description of the timeline and scale of the nitazene epidemic’s appearance and evolution in Estonia. To understand the response to the epidemic, data for service delivery was obtained from the National Institute for Health Development (NIHD), the central provider of harm reduction and treatment services in the field of illegal drugs in Estonia that includes an overview of the volume of services offered through its national monitoring system.

### Qualitative data collection

We conducted semi-structured interviews with ten individual nitazene users in Tallinn and Narva to gain a deeper understanding of the lived experiences of such use. These interviews, conducted between October 3rd and 28th, 2024, were designed to complement and contextualize the findings of the administrative data review, with a particular focus on the use of nitazenes in Estonia. The face-to-face interviews by trained psychologists of harm reduction services, lasting ∼40 minutes each, were audio and tape-recorded. An interview guide was created in both Estonian and Russian languages and consisted of 26 questions covering the following themes: previous experience with opioid use; specific experiences in the use of nitazenes; methods of obtaining nitazenes; perceived health consequences of using nitazenes; and harm reduction strategies employed (∼1).

Study participants were identified by harm reduction service providers in Tallinn and Narva, representing the two primary areas of illicit drug use in Estonia. Interviewees were selected from users who reported having used synthetic opioids with effects different from fentanyl in the past 3 years, or who identified the street names of the nitazenes they were using. The data collected were analysed using framework analysis [18], a qualitative method that allows for a systematic and transparent approach to analysing textual data.

To provide insights into the strategies, challenges, and on-the-ground experiences of those directly involved in addressing the crisis, the first author also collected structured information from first responders and treatment providers through personal communication via email and phone (*n* = 5). Healthcare workers were asked four questions about how the arrival of nitazenes to Estonia affected their work; whether conventional approaches were working; whether they had accurate information available; and whether they felt there was a lack of resources to adequately respond.

The study was approved by the Ethics Committee for Human Research of the National Institute for Health Development (nr 1379, 07.10.2024).

## Results

### Emergence and evolution of nitazenes in Estonia

#### Estonian forensic science data

**Figure 1.**
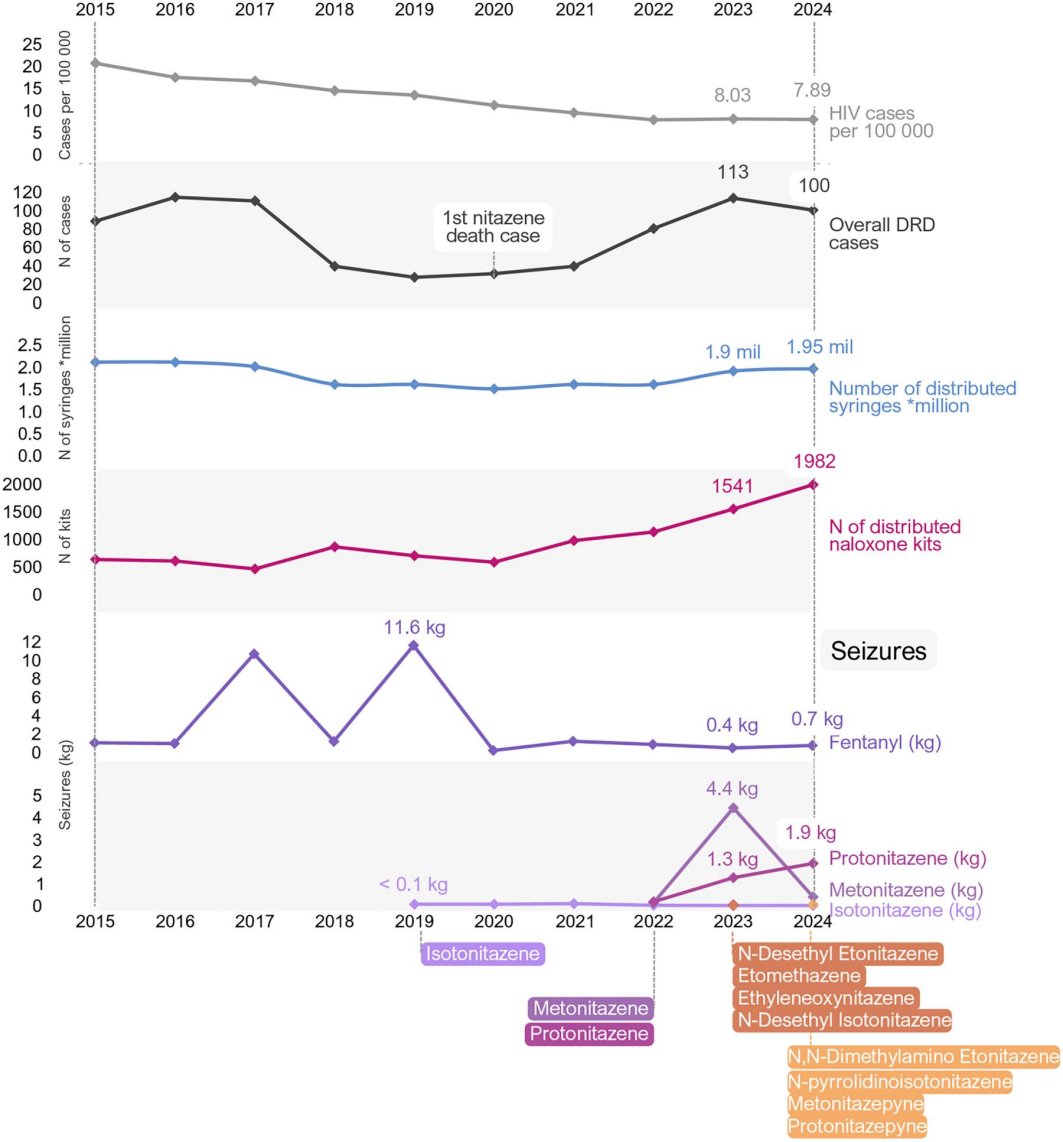
The timeline of the emergence and evolution of nitazenes in Estonia 2015–24. Sources: Estonian Forensic Science Institute, 2025; Causes of Death Registry, 2025; National Institute for Health Development (NIHD), 2025; Estonian Health Board, 2025 (Supplementary Table S1).

The timeline for the emergence and evolution of nitazenes in Estonia is depicted in Fig. 1. The first official detection occurred on October 11, 2019, when the Estonian Forensic Science Institute (EFSI) reported the seizure of 3.6 g of isotonitazene [19]. EFSI had the capacity, based on international cooperation and the availability of reference samples, to detect nitazenes beforehand, but this was the first detection. Notably, Estonia was the first country in the European Union to identify ethyleneoxynitazene, highlighting the advanced analytical capabilities of its forensic laboratory [19].

Although there was a large-scale seizure of fentanyl in 2019, the substance was uncommon on the Estonian drug market. Seizures of isotonitazene continued to a limited extent in 2020–21 (Fig. 1). In 2022, based on seizure statistics, highly potent metonitazene and protonitazene reached the Estonian drug market, which have since become the predominant opioids. The increased presence of nitazenes on the Estonian drug market was not only demonstrated by the quantities seized, but also by the number of seizures of different nitazenes, which increased from 53 cases in 2022—132 cases in 2023 and 86 in 2024. Additionally, N-desethylisotonitazene emerged and supplanted isotonitazene, mirroring trends observed with other nitazene analogues in 2023 and 2024 (Fig. 1). In 2022–23, most nitazene seizures involved a single nitazene compound, and in 2024 mostly in mixtures. Among the detected mixtures, the most prevalent combination has been metonitazene and protonitazene. In one isolated case, a mixture of protonitazene, metonitazene, and N-desethyl Isotonitazene was identified. In 2023–24, many novel nitazenes were detected in a limited number of cases (Fig. 1).

Chemical analysis of metonitazene and protonitazene samples from 2022 to 2024 identified the presence of other psychoactive substances, including methadone, amphetamine, cocaine, fentanyl, carfentanil, diphenhydramine (DPH), bromozolam and, in a concerning trend, xylazine, the presence and dangerous combination of which has occurred in a few cases over recent years [20, 21].

#### Analysis of the residual content of used syringes and wastewater

**Figure 2.**
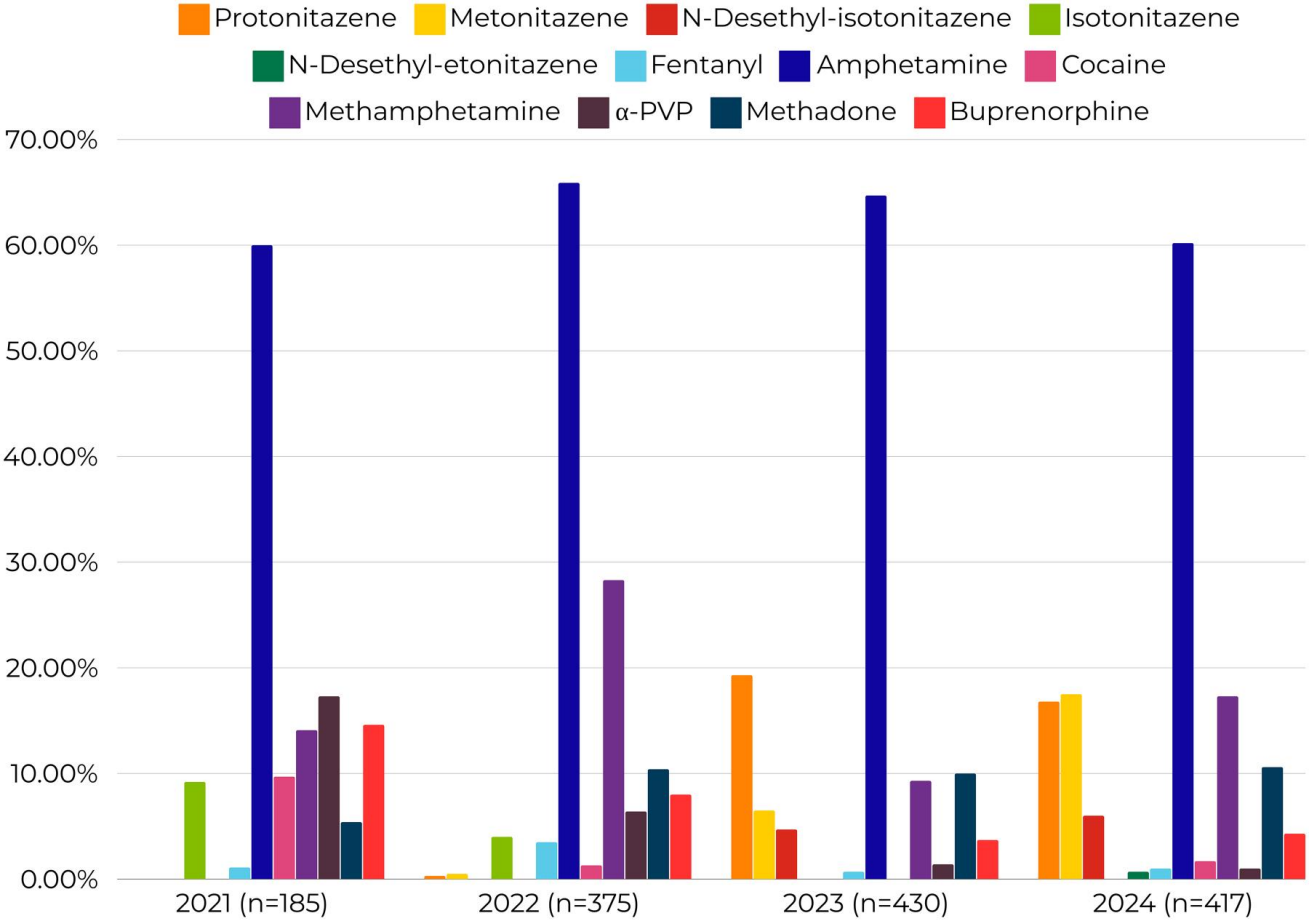
The main substances in syringes 2021–24 (%). Source: Syringe residue study data 2021–24 [22–24].

To obtain objective chemical data on injection practices, syringe residue studies were conducted from 2021 onwards (Fig. 2). A liquid chromatography quadrupole mass spectrometer with a time-of-flight detector and, in some cases, a gas chromatography mass spectrometer was used for analysis [22–24]. Although amphetamine has been the dominant illicit drug injected, studies have revealed a concerning increase and geographic spread in the use of nitazenes over the years. In 2021, a pilot study in two Estonian regions detected isotonitazene in 9% of syringes compared to 1% of syringes containing fentanyl [22]. In 2022, the study expanded to include samples from all harm reduction services (*n* = 21) across Estonia. While isotonitazene remained the most prevalent nitazene, the emergence of protonitazene and metonitazene was observed, indicating diversification within the nitazenes market. In 2022, 3.5% of syringes analysed contained fentanyl [23].

In 2023, the syringe residue study found that isotonitazene was entirely replaced by N-Desethyl-isotonitazene and only four syringes contained fentanyl (Fig. 2). Compared to 2022, the share of syringes containing nitazenes increased sharply from 2023 (6% vs 22%). The geographic distribution of injected nitazenes also expanded, spreading from the capital regions to other areas over the two study years. The most frequently detected nitazenes in syringes in 2023–24 were protonitazene, followed by metonitazene. Notably, 2024 differed from 2023 by the presence of mixtures of metonitazene and protonitazene in syringes. N-Desetyl-etonitazene was detected for the first time in 2024 [23, 24].

The prevalence of nitazene use is further supported by a wastewater study which qualitatively detected nitazenes in several routine wastewater samples in the capital region in 2023 [25].

#### Estonian causes of death registry

The first fatalities related to nitazenes were documented in 2020, with a limited number of additional cases occurring in 2021 (Table 1). Notably, fentanyl-related deaths during the period 2019–21 remained relatively low compared to the period 2002–17 [12].

**Table 1.** Demographic characteristics of nitazene-involved overdose deaths during 2019–24. (Estonian residents)

Characteristic	2019	2020	2021	2022	2023	2024
Total number of DRD	27	31	39	80	113	100
Rate per 100 000 population	2.04	2.33	2.93	6.01	8.27	7.32
Mean age	36.5	36.2	40.2	38.0	36.8	38.3
Men %	74%	84%	74%	80%	76%	82%
Death cases related to nitazenes	0	3	4	30	57	42
inc. Metonitazene				7	28	31
inc. Isotonitazene		3	4	4		
inc. Protonitazene				22	37	35
inc. N-Desethyl isotonitazene					2	2
Mean age		34.3	29.5	40.2	38.4	38.2
Men (%)		67%	75%	93%	84%	86%

Source: Estonian causes of death registry, 2025.

Using data from the Estonian causes of death registry (Table 1), there were a total of 136 fatal drug overdose cases involving nitazenes among Estonian residents during 2019–24. In 2019–21, there were few cases of drug-related death (DRD) per year related to nitazenes; from 2022, cases began to increase rapidly, forming more than half of DRD cases in 2023 and almost half of DRD cases in 2024.

Postmortem toxicological analysis of 136 deaths related to nitazenes (2019–24) revealed a high prevalence of polydrug use. While 22 cases involved a single nitazene, the majority indicated multiple substances. Among the most frequently co-occurring substances were benzodiazepines (62 cases), particularly diazepam, alprazolam, and clonazepam. Stimulants were also prevalent, with 34 cases involving amphetamine and 18 involving cocaine. Notably, 39 cases involved multiple nitazenes, most commonly protonitazene and metonitazene. The presence of xylazine, a dangerous combination [20, 21], was detected in four cases. Naloxone was detected in 15 cases (11.2%), mostly in the years 2023–24. The DRD cases without the presence of nitazenes mostly involved polydrug use where there was a combination of stimulant (amphetamine, cocaine) together with methadone or other opioid (tramadol, oxycodone) and many benzodiazepines and antidepressants.

#### Qualitative interviews with people who use nitazenes

The 10 interviewees had an average age of 39.8 years (range: 27–48 years), with an equal representation of men and women. Five interviews were conducted in East-Virumaa and five in the Tallinn region. Most participants reported initiating drug use around the ages of 15–16 years, transitioning to opioids around the age of 18. The average duration of drug use among interviewees was 21 years. While some participants mentioned starting with cannabis or amphetamines, they rapidly transitioned to injecting available opioids, including poppy liquid, heroin, fentanyl, and carfentanil. All interviewees had initiated the use of nitazenes within the past 1–3 years.

Patterns of nitazene use varied among participants, ranging from daily use to heavy episodic use with month-long periods of abstinence interspersed with periods of intense use lasting up to 1 week. The frequency of daily use ranged from once per day to every 2–3 hours, with the exception being those who smoked nitazenes who reported hourly use. Nine-out-of-ten interviewees reported injecting nitazenes intravenously, while one smoked the substance.

Nitazenes on the Estonian drug market were typically encountered as a wet powder in various colours, including white, yellow, brown, rose, and beige. The common understanding among people who use drugs was that one dose (‘фитюлька’) of a nitazene costs €25, with 1 g yielding ∼20 doses. Most participants expressed a preference for carfentanil, indicating that the shift to the use of nitazenes was driven by market availability. Table 2 presents a detailed breakdown of themes and sub-themes derived from qualitative interviews, illustrating the experiences and perspectives of nitazene users.

**Table 2.** Themes and sub-themes from qualitative interviews with nitazene users, Estonia (2024)

Main theme	Corresponding sub-themes	Quote
Unintentional first-time use and continued lack of information about the substance	Unintentional first-time use Sold under various street names Users crave synthetic opioids without knowing the exact substance Market-driven use, not conscious preference	*‘First it was ’собака’, then the knowledge of nitazenes arrived’. (N7)* *‘I don’t know what is nitazene, but I use “собака. (dog)”’. (N3)* *‘They sell it under fentanyl,* “тяжелый” (*heavy); nitazene is not mentioned’. (N4)*
Potent and transient effect of nitazenes	Effects: stronger, faster, shorter-lived than fentanyl Causes ‘nodding out’ memory impairment Compulsive redosing, diminishing returns	*‘When you take it, you fall asleep, which is not sleep because you perceive everything around you, but do not feel it correctly’. (N1)* *‘The effect of nitazenes is strong, fast, sudden and short-lived. There is no point in re-dosing. No desired effect. Differs from fentanyl in that’. (N3)* *‘Fentanyl leaves some control over consciousness, nitazenes don’t’. (T6)*
More severe and complex withdrawal symptoms	More severe/complex withdrawal than fentanyl Methadone often insufficient Persistent symptoms (e.g. cold, sweating, pain)	*‘There is a quick tolerance, I feel bad when not using it, like the flu…I twitch and shake…There are also episodes of depression. Anger at yourself. Cold…’. ‘The withdrawal wasn’t as bad with the fentanyl’. (N2)* *‘With fentanyl use, methadone removed all the withdrawal symptoms, but with nitazenes, some of the symptoms remain for days, like feeling of cold, sweating, pain and tremors’. (T7)*
Perceived ineffectiveness of naloxone and need for higher doses	Perceived naloxone ineffectiveness Multiple/alternative naloxone doses required Injectable naloxone perceived more effective	*‘And one naloxone isn’t enough anyway with nitazenes anymore. And this injectable version of naloxone is more effective, you can’t dose the nasal one, it doesn’t help’. (T10)* *‘My friend’s husband overdosed under the bush. We did two nasal doses, no effect, then the ambulance came and gave more naloxone, also nothing, then they injected something into the vein, then after 10 minutes he woke up’. (T9)*
Harm reduction strategies employed	User-initiated dose reduction/safety Avoiding solitary drug consumption Attempting to assess the quality of the substance	*‘The principle of safety is still that you use less. There is no other way. If you want to live, you don’t do everything at once’. (N1)* *‘If it doesn’t seem to be the right stuff, use it with dots. I take 6 dots, divide and test’. (T9)* *‘I don’t use alone, I'm afraid. You put the syringe in the vein, push in one amount, half of the syringe…you wait, the syringe stays in the vein. You understand that it has an effect and a good effect, then you take another half’. (N1)*
Lack of reliable information	Incomplete dealer infomation Relies on personal/informal networks	*‘Dealers say use it carefully, it is not fentanyl’. (T6)* *‘In fact, it doesn’t matter what is said, if you need a substance, you take and inject whatever is available!’ (T10)* *‘The time of carfentanil was the last time when dealers talked about the substance at all, then all information about it ended’. (N5)*

#### Information from health care and first respondents

The emergence of metonitazene and protonitazene in 2022 directly led to a rapid surge in overdose cases in Tallinn compared to 2021, placing immediate and significant strain on the Tallinn Medical Emergency Service (366 vs 605 cases). The first weeks of the nitazene epidemic of May and June 2022 kept the first responders in Tallinn on constant alert. A critical observation was that nitazene overdoses presented severe clinical manifestations, necessitating significantly higher doses of naloxone compared to typical fentanyl overdoses. The ambulance service described patients who were stabilized with naloxone but later still relapsing. This highlights the extreme potency of these novel synthetic opioids. The ambulance service lacked readily available information on drug market trends, the extent of the problem and the long-term effects. The ambulance services sought information from other authorities about the potent substance on the market. This period once again highlighted the need for a functioning national early warning system.

Soon nitazenes also became a challenge in treatment and withdrawal management. Preliminary insights from treatment providers indicated that an individual’s dependence on nitazenes may require higher doses of opioid substitution therapy. Furthermore, residential treatment centres reported increased difficulty in managing withdrawal symptoms and observed lower treatment adherence among this population.

#### Harm reduction services

Data from harm reduction services indicate a steady increase in the number of clients, service utilization, and syringes distributed since 2021 (Fig. 1). Estonia’s prescription-only take-home naloxone (THN) programme started in 2013 with the distribution of prefilled syringe kits. Since 2018, it has transitioned to nasal spray, but prefilled syringes are also available [26]. While Estonia’s THN programme was initially correlated with a decrease in overdose deaths [14], this trend shifted in 2022, coinciding with the emergence of potent nitazenes, including protonitazene and metonitazene (Fig. 1).

Since 2022, Estonian harm reduction services have been advised by the NIHD to distribute multiple THN kits and to train users to administer multiple doses of naloxone if needed. Since 2024, nasal naloxone has been provided to Estonian police officers who are often the first responders in drug overdose cases [27].

## Discussion

This study offers a nationwide examination of the emergence and impact of nitazenes in Estonia. Our findings, drawn from administrative statistics, surveillance data, and qualitative interviews, indicate a growing presence of nitazenes since 2022, supported by syringe residue studies, drug-related death statistics, and seizure data. These substances frequently appear to be sold on the illicit drug market with limited or no information regarding their content, potentially increasing risks for users who may be unaware of their true potency and dangers. The rise of nitazenes seems linked to a surge in drug-related deaths, particularly among individuals with prior experience using other opioids such as fentanyl.

The nitazene epidemic in Estonia, characterized by a rapid surge in drug-related deaths and the emergence of highly potent compounds, mirrors alarming trends observed in other regions, particularly in Europe and North America. Similar to findings in the UK, Latvia, and the USA, nitazenes are exacerbating existing opioid crises and linked to increased fatalities [7, 28]. However, Estonia’s distinct trajectory, transitioning from a prolonged fentanyl epidemic to a nitazene-dominant landscape, offers a unique perspective as an early indicator for shifting synthetic opioid trends in Europe. Furthermore, the local insights regarding users’ perceptions of methadone treatment primarily for withdrawal management and the specific application of syringe residue and wastewater analysis provide context-specific nuances to the broader international understanding of this escalating public health crisis.

### Nitazenes in Estonia: underestimated threat and rising fatalities

Despite its prior experience with fentanyl, Estonia has faced challenges in adapting swiftly to the emergence of nitazenes in 2019. These potent synthetic opioids, often sold without clear identification, appear to have infiltrated the drug market, posing significant risks, especially to users accustomed to fentanyl. A notable association is observed between nitazenes and the increase in drug-related deaths in Estonia since 2022. The initial lower potency of isotonitazene [29] might have contributed to an underestimation of the nitazene threat, potentially delaying early awareness and response. This delayed recognition, coupled with the increasing detection of nitazenes, seems to have contributed to a rise in drug-related deaths, particularly among experienced individuals who use drugs. Given the reliance on a 2015 estimate for the population of people who inject drugs in Estonia, ∼2600 opioid users may face exposure to nitazenes in the current drug market [30, 31]. The qualitative interviews suggest that the low self-reported use of nitazenes among people who inject drugs may reflect a lack of awareness among users about the specific substances they are consuming, a discrepancy noted in other contexts with changing drug markets [32].

### Frontline challenges and surveillance gaps

The emergence of nitazenes has placed considerable strain on emergency healthcare providers. Frontline workers often encounter overdose cases with limited immediate knowledge about the novel drug’s specific toxicology and optimal use of opioid antidotes. This potential information gap could hinder effective response efforts and affect the safety of both responders and individuals experiencing overdose [33].

Moreover, the nitazene epidemic highlights existing gaps in surveillance systems. Traditional monitoring approaches, often relying on administrative statistics and surveys, may not always detect emerging drug trends in a timely manner. Such delays could impede the implementation of swift and targeted interventions. To maintain current information on the illicit drug scene, exploring additional information sources is indicated [14]. Estonia’s experience with innovative methods, such as wastewater and syringe residue analysis implemented as surveillance tools, offers objective, near real-time data on drug trends. Drug checking services could also serve as another channel for continuous monitoring of drug market dynamics. Evidence suggests these services can help mitigate substance use risks, including overdose, by enabling users to test their supply [34], and appear responsive to changes in drug markets, including the emergence of novel psychoactive substances and highly potent compounds [35–37].

### Harm reduction and supply control: a dual approach

Estonia’s response to the nitazene epidemic appears to highlight both strengths and areas for further development in its harm reduction and treatment strategies. While harm reduction services, including naloxone distribution and needle exchange programmes, are generally available and utilized, the absence of drug consumption rooms may limit opportunities for preventing overdoses [38] and improving health outcomes for people who use drugs. Although naloxone is generally effective in reversing nitazene-involved overdoses, observations suggest that multiple doses are often required, and some users express uncertainty regarding its immediate efficacy [4, 39]. The increased detection of naloxone in post-mortem toxicology in 2023–24 might indicate broader naloxone use but could also point to increasing dosage requirements for effective overdose reversal in cases involving nitazenes and polydrug use.

To contribute to reducing the supply of nitazenes, proactive action is suggested. Nitazenes pose a notable challenge due to their potentially inexpensive production, readily available precursors, and ease of transport [5, 33]. These factors, combined with their potent psychoactive effects and potential for high illicit profits, may make them attractive to unauthorized manufacturers and distributors. The emergence of novel psychoactive substances like nitazenes appears to necessitate a proactive and adaptive approach to drug legislation and enforcement. This could include continuous monitoring of the drug market, swift scheduling of new substances, and international collaboration to manage precursor chemicals and disrupt trafficking networks [33].

## Conclusion

The nitazene epidemic in Estonia exemplifies the dynamic nature of the drug market and underscores the need for adaptable and responsive harm reduction and treatment strategies. While current knowledge about nitazenes remains limited, the broader global drug market trends and the 2022 poppy ban in Afghanistan highlight the ongoing need for evidence-based information on these novel synthetic opioids. Also, close monitoring of the impact of China’s July 1, 2025, nitazene control measures on their use and availability will be essential [40]. As nitazenes continue to emerge and become more prevalent, countries are encouraged to develop and refine effective response strategies to help prevent further loss of lives.

### Limitations

While this study relied on administrative data and a limited number of qualitative interviews, it aims to provide valuable initial insights into the nitazene epidemic in Estonia. Administrative data, though potentially limited in scope, offers a broad overview of nitazene trends. Additionally, the qualitative interviews, while small in number, provide unique first-hand accounts of experiences and challenges faced by people who use drugs. This study represents an important initial step in understanding the nitazene epidemic in Estonia and aims to lay the groundwork for future research.

## Supplementary Material

ckaf160_Supplementary_Data

## Data Availability

The data underlying this article will be shared upon reasonable request to the corresponding author. Key points The use of nitazene has increased between 2019 and 2024 in Estonia. Drug-related deaths have more than doubled since 2022, with nitazenes causing almost half of all cases of drug-related deaths. Nitazenes are often sold on the drug market, usually with little or no information for the user about the substance being sold. The use of harm reduction services (including take-home naloxone) has increased since 2022. The nitazene epidemic in Estonia serves as a critical reminder of the dynamic nature of the drug market and the need for adaptable and responsive harm reduction and treatment strategies.
