# Supplementary material for: The nitazene epidemic in Estonia: a first report
Source: Eur J Public Health. 2025 Sep 23;35(6):1233–40. doi: 10.1093/eurpub/ckaf160 (PMC12707477; doi:10.1093/eurpub/ckaf160)
Supplement: ckaf160_Supplementary_Data [file ckaf160_supplementary_data.docx]

**Supplementary files**

**Table S1** Description of data sources.

|  | Causes of Death Registry^[[1]](#endnote-1)^ | Estonian Forensic Science Institute^[[2]](#endnote-2)^ | Estonian Health Board^[[3]](#endnote-3)^ |
| --- | --- | --- | --- |
| Data | Drug-related death cases | Illicit drug seizure statistics  Syringe and wastewater residue studies | Infectious disease surveillance (HIV) |
| Primary objective | The purpose of the register is to collect and process data on causes of death for the development of national social policy, to assess the composition and health status of the population, and to plan preventive activities in the social and health fields. | Records seizures of illegal substances, providing insight into drug trafficking patterns and law enforcement activities | Monitor the prevalence, incidence, and trends of HIV infections over time,  ICD-10 |
| Coverage | National /Estonia | National /Estonia | National /Estonia |
| Population | General / population based | Specific incidents such as drug seizures | General / population based |
| Data collection | Data is collected via the X-road data exchange layer from the health information system, the Estonian Population Register, and the pregnancy information system for deaths in the perinatal period. | Electronic data reports of seizure events  Syringe and wastewater samples | Electronic data reports of passive surveillance reports (health care providers, laboratories) |
| Frequency of data update | Continuous | Continuous | Continuous |
| Data Structure and Variables* | Causes and circumstances of death. Drug-induced deaths (i.e. accidental and undetermined intent poisonings, suicide, aspiration and addiction caused by narcotic drugs). | Type and quantity of drugs seized | Number of new (diagnosed) cases |
| Data owner | Ministry of Social Affairs | Ministry of Justice | Ministry of Social Affairs |
| Period covered* | 2015─2024 | 2015─2024 | 2015─2024 |

*For this review

1. https://www.tai.ee/et/statistika-ja-registrid/surma-pohjuste-register [↑](#endnote-ref-1)
2. https://www.ekei.ee/en [↑](#endnote-ref-2)
3. <https://www.terviseamet.ee/en/communicable-diseases/statistics/communicable-disease-bulletins>

   **Appendix 1.**

   **Semi-structured interview guide**

   (NB! The subject's experience of using nitazene can be both current and previous)

   **Questions on nitazene use**

   **1. Some questions about the subject**

   - Gender
   - Age
   - Drug use experience

   **2. Experience with opioid use**

   - When did you start using opioids?
   - What was the first opioid you used?
   - When was your first exposure to nitazene?
   - Was it conscious use or did you use a new opioid under the name of another already known substance?
   - If not, under what name were nitazene sold to you?

   **3. Information on nitazene use**

   - Have you consciously purchased nitazene yourself?
   - Under what name are nitazene sold/have they been sold on the Estonian drug market? (including street names)
   - How does the immediate effect of nitazene differ from the effect of other opioids you have used?
   - What feeling/state does using nitazenes cause?
   - How often do you use nitazenes?
   - What is your usual dose of nitazenes?
   - In what way do you mainly use nitazenes?
   - Do you also use other drugs/intoxicants in parallel?
   - What is the main reason for using nitazenes?

   **4. Obtaining nitazenes (NB**! If respondent wants to talk, if not, it is understandable, and you will continue with other topics)

   - How much does a dose of nitazene cost on the street?
   - Where do you usually get nitazenes?
   - How is the substance delivered to you?

   **5. Health effects of using nitazenes and harm reduction measures**

   - Have you experienced any immediate and/or later negative health effects related to using nitazenes?
   - Do you know how dangerous/potent nitazenes are?
   - What is your main source of information related to nitazenes?
   - Do sellers/dealers explain the nature of the substance and the specifics of use in the case of nitazene?
   - Do you have any activities/routines in place to make nitazene use safer?
   - Do you have any clever tricks/techniques on how to assess the quality of nitazene yourself?
   - Do you/your companions have a naloxone kit (nasal spray) to take home and can you/can help in case of an overdose?
   - Have you come across nitazene overdoses (you have overdosed yourself, friends, loved ones, etc.)?
   - Are you aware of any approaches/services elsewhere in the world that would help drug users cope better in the current situation?
   - If so, do you think that Estonia should have any additional services for drug users that would help opioid users cope better? (the possibility of testing the substance/its strength before use, safe-supply or legal and regulated availability of narcotics for long-term opioid users, etc.).

   **First respondent topics** (information collected via e-mail and phone conversations):

   1. When you think back to the emergence of metonitazene and protonitazene in the Estonian drug market (2022), how did it affect your work?
   2. Did you have to change anything in your usual work procedures/approaches?
   3. Was the necessary support/information available to you about what is happening in the drug market and what is being used?
   4. Did you feel at the time that you were missing something to better help the victims of overdose? If yes, what was it?

   [↑](#endnote-ref-3)
